# Scavengers as Prospective Sentinels of Viral Diversity: the Snowy Sheathbill Virome as a Potential Tool for Monitoring Virus Circulation, Lessons from Two Antarctic Expeditions

**DOI:** 10.1128/spectrum.03302-22

**Published:** 2023-05-25

**Authors:** Gabriel Zamora, Sebastian Aguilar Pierlé, Johana Loncopan, Loreto Araos, Francisco Verdugo, Cecilia Rojas-Fuentes, Lucas Krüger, Aldo Gaggero, Gonzalo P. Barriga

**Affiliations:** a Laboratory of Emerging Viruses, Virology Program, Institute of Biomedical Sciences, Faculty of Medicine, Universidad de Chile, Santiago, Chile; b Inorevia, Pepinière Paris Santé Cochin, Paris, France; c Laboratory of Environmental Virology, Virology Program, Institute of Biomedical Sciences, Faculty of Medicine, Universidad de Chile, Santiago, Chile; d Instituto Antártico Chileno, Punta Arenas, Chile; e Fundación Instituto de Biodiversidad de Ecosistemas Antárticos y Subantárticos, Las Palmeras, Ñuñoa, Santiago, Chile; f Facultad de Ciencias de la Salud, Programa Magister en Ciencias Químico Biológicas, Universidad Bernardo O’Higgins, Santiago, Chile; University of California, San Diego

**Keywords:** surveillance, emerging viruses, Antarctica, snowy sheathbill, zoonosis

## Abstract

Antarctica is a unique environment due to its extreme meteorological and geological conditions. In addition to this, its relative isolation from human influences has kept it undisturbed. This renders our limited understanding of its fauna and its associated microbial and viral communities a relevant knowledge gap to fill. This includes members of the order Charadriiformes such as snowy sheathbills. They are opportunistic predator/scavenger birds distributed on Antarctic and sub-Antarctic islands that are in frequent contact with other bird and mammal species. This makes them an interesting species for surveillance studies due to their high potential for the acquisition and transport of viruses. In this study, we performed whole-virome and targeted viral surveillance for coronaviruses, paramyxoviruses, and influenza viruses in snowy sheathbills from two locations, the Antarctic Peninsula and South Shetland. Our results suggest the potential role of this species as a sentinel for this region. We highlight the discovery of two human viruses, a member of the genus *Sapovirus* GII and a gammaherpesvirus, and a virus previously described in marine mammals. Here, we provide insight into a complex ecological picture. These data highlight the surveillance opportunities provided by Antarctic scavenger birds.

**IMPORTANCE** This article describes whole-virome and targeted viral surveillance for coronaviruses, paramyxoviruses, and influenza viruses in snowy sheathbills from the Antarctic Peninsula and South Shetland. Our results suggest an important role of this species as a sentinel for this region. This species’ RNA virome showcased a diversity of viruses likely tied to its interactions with assorted Antarctic fauna. We highlight the discovery of two viruses of likely human origin, one with an intestinal impact and another with oncogenic potential. Analysis of this data set detected a variety of viruses tied to various sources (from crustaceans to nonhuman mammals), depicting a complex viral landscape for this scavenger species.

## INTRODUCTION

Antarctica is populated by a diverse and unique fauna with its associated microorganisms; however, our knowledge of the microbial and viral diversity on this continent, including pathogens with zoonotic potential, remains limited (1, 2). The region’s geographic isolation has been associated with viral paucity and probable endemic evolution (3). Currently, a small number of viral species have been described in Antarctic fauna, mostly through studies of penguin populations and a few screens of additional bird species (2–5). Little is known about Antarctic fauna members of the order Charadriiformes and the viruses that they carry. Birds of this order, which includes polar skuas (*Catharacta* spp.), Antarctic terns (Sterna vittata), and snowy sheathbills (Chionis albus), breed on the Antarctic Peninsula during the Antarctic summer and then migrate northward during the nonbreeding season (6). This implies the movement of the microorganisms (and potential pathogens) that they harbor between Antarctica and South America (7). Such species are an interesting prospect to provide us with a detailed picture of the viral diversity in Antarctica. Snowy sheathbills are opportunistic predator/scavenger birds distributed on Antarctic and sub-Antarctic islands (2). This species breeds near penguin colonies (6). The isolation and extreme climate of its breeding grounds made snowy sheathbills flexible enough to take advantage of various food sources. These include sea algae, crustaceans, and placentas and carcasses of marine mammals (2). Their frequent contact with different bird and mammal species, in addition to their foraging/scavenging behavior, makes them an appealing species for surveillance studies due to their high potential for the acquisition and transport of viruses (8, 9).

In this study, we performed targeted viral surveillance for coronavirus, paramyxovirus (PMX), and influenza virus complemented by RNA virome characterization in snowy sheathbills from the Antarctic Peninsula and South Shetland. Our results suggest a role of this species as a potential sentinel for this region. This species’ RNA virome showcased a diversity of viruses likely tied to Antarctic fauna. We highlight the discovery of two viruses of likely human origin, a member of the genus *Sapovirus* GII and a gammaherpesvirus (Epstein-Barr virus), and additional viruses from marine mammalians. The path by which these viruses were introduced into Antarctica remains to be resolved. These data highlight the surveillance opportunities provided by Antarctic scavenger birds.

## RESULTS

### Surveillance for avian viruses previously detected in Antarctica.

**FIG 1:**
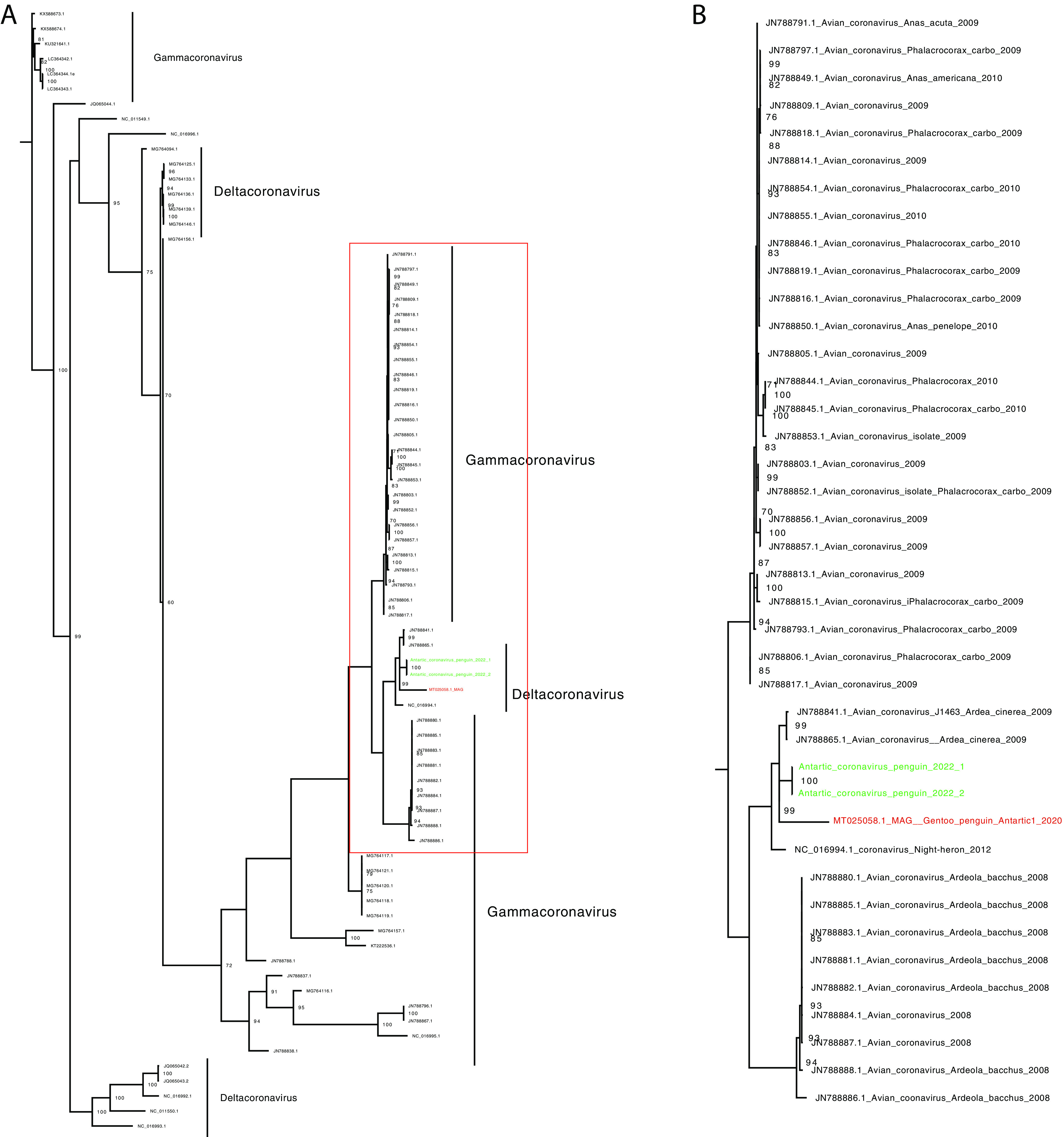
Phylogenetic tree including members of the gamma- and deltacoronavirus genera. Sequence comparison was performed using 78 sequences from the gamma- and deltacoronavirus genera; the coronavirus isolated in our study (shown in green) appears to be closely related to a deltacoronavirus sequenced in 2009 and clusters with a sample of a partially sequenced virus from a gentoo penguin (shown in red). Phylogenetic distances were calculated using the GTR method. A bootstrap analysis with 1,000 replicates was applied, and the numbers indicate bootstrap values. (A) Complete phylogenetic tree; (B) zoomed-in view of the Antarctic sequence clade (green).

**FIG 2:**
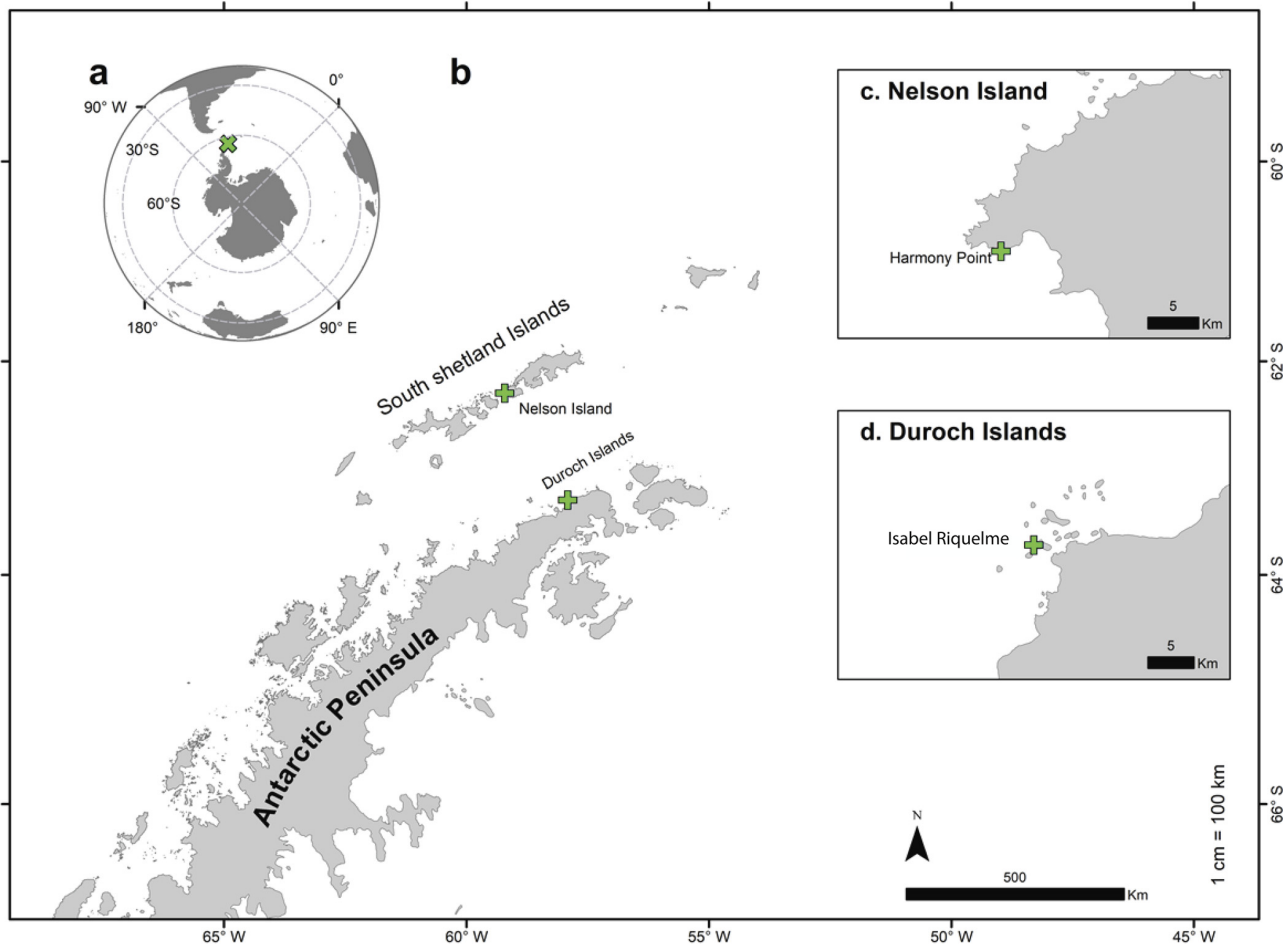
Locations of sampling sites for snowy sheathbills in the Antarctic Peninsula’s Harmony Point, Nelson Island, and Duroch Islands. Shown are the positions of sampling sites at the Southern Hemisphere scale (a) and the Antarctic Peninsula scale (b), the location of Harmony Point on Nelson Island (c), and the location for Kopaitic Island/Isabel Riquelme Islet on the Duroch Islands (d). Samples from locations in panel c were collected in 2020, and samples from locations in panel d were collected in 2022.

First, we attempted to gauge the circulation of economically/ecologically relevant viruses in our sampled population. For this, we targeted previously reported avian viruses in Antarctica. More specifically, we targeted viruses with known impacts on poultry or wild fauna, including coronaviruses, paramyxoviruses, and influenza viruses (Table 1). Eight samples positive for coronavirus were identified during Chilean Antarctic Expedition 57 in 2020 (ECA57/2020) on Nelson Island, and 6 positive samples were identified during ECA58/2022 around Isabel Riquelme Islet. The samples were positive using degenerate primers. It should be noted that all samples were positive for the Antartic1 replicase gene. Furthermore, one coronavirus isolate was partially sequenced for each year (Table 1). The samples clustered with a deltacoronavirus sequence from a gentoo penguin (Fig. 1). Next, eight samples positive for paramyxovirus were detected during ECA57 (Nelson Island) (Fig. 2), and all samples were positive for avulavirus 18 by reverse transcription-PCR (RT-PCR) (Table 1). Twelve samples were positive using panparamyxovirus primers during ECA58 (Isabel Riquelme Islet) (Fig. 2). Samples from this expedition were positive for avulaviruses 18 and 19 by RT-PCR (Table 1). In total, we identified 5 environmental samples positive for avulavirus 18 and 6 snowy sheathbill samples positive for avulavirus 19 (total of 11 samples). Only one sample was negative using all tested avulavirus primer pairs (avulavirus 17, 18, or 19 or Newcastle disease virus [NDV]), suggesting a probable novel sequence (Table 1). Two environmental samples from ECA58/2022 were coinfected with avulaviruses 18 and 19 (Table 1).

**TABLE 1:** Pools positive by pancoronavirus and panparamyxovirus primers per expedition^*a*^

Primer type	No. of samples					
	ECA57			ECA58		
	Environmental	Snowy sheathbill	Positive (total)	Environmental	Snowy sheathbill	Positive (total)
Pan-CoV	6	2	8 (25)	3	3	6 (21)
Viral isolated	1	0	1 (25)	1	5	6 (21)
Pan-PMX	5	1	6 (25)	6	6	12 (21)
AV-18	5	1	6 (25)	5	6	11 (21)
AV-19	0	0	0 (25)	2	0	2 (21)
Viral isolated	0	0	0 (25)	0	3	3 (21)

a Note that the samples were analyzed by RT-qPCR against influenza virus and by RT-PCR against NDV. Avulavirus 17 was also tested for; however, no positive samples were detected. AV-18, avulavirus 18; CoV, coronavirus; PMX, paramyxovirus.

**FIG 3:**
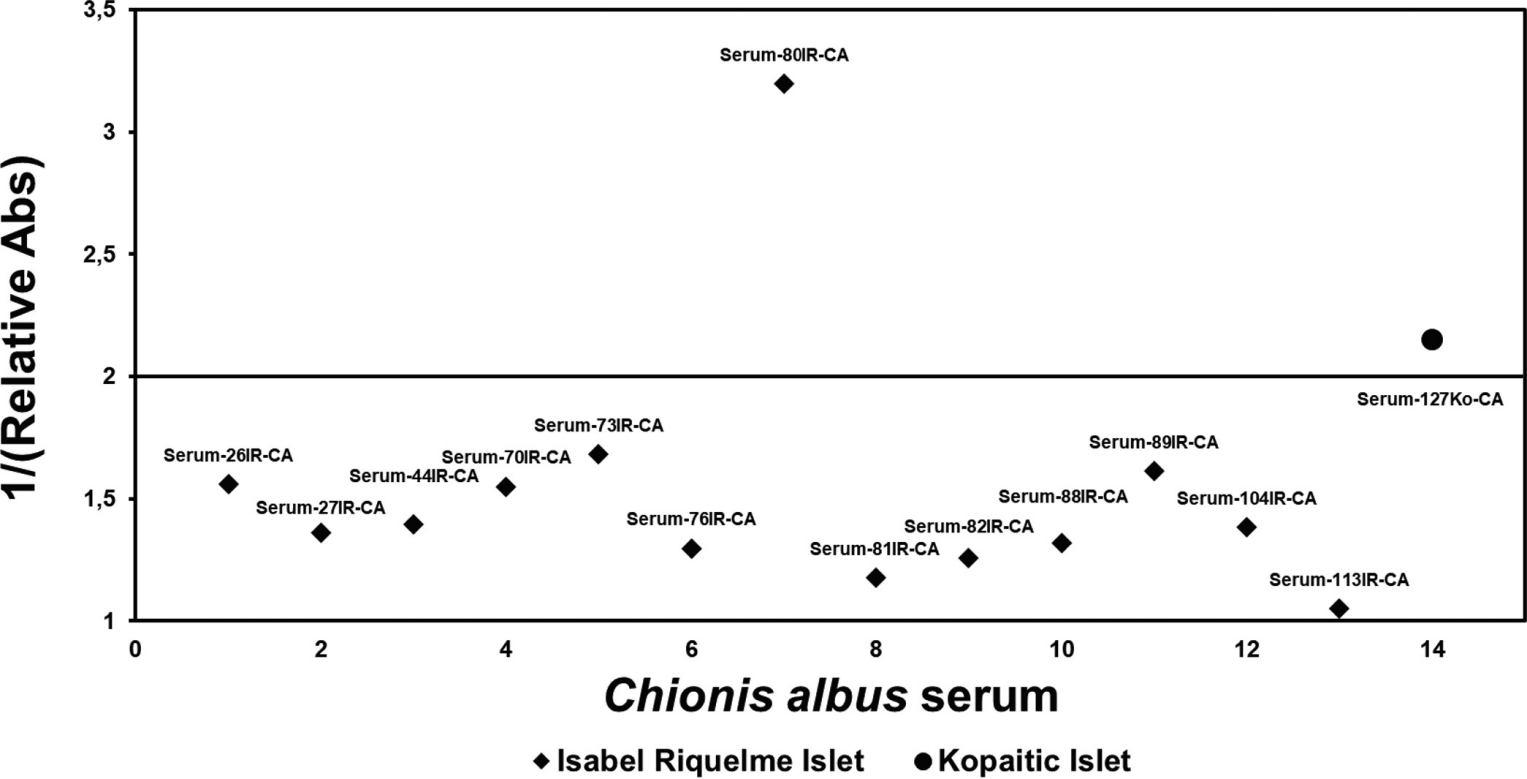
Avian influenza virus (AIV) seroprevalence in snowy sheathbills. Shown are data from competitive nucleoprotein (NP) ELISAs of serum samples obtained from snowy sheathbills. Rhombus dots display Isabel Riquelme Islet samples, and circles display Kopaitic Island samples. Overall, there was a 14% AIV seroprevalence. Abs, antibodies.

To show influenza virus circulation, we screened samples from 14 snowy sheathbills from 2 locations (Kopaitic Island and Isabel Riquelme Islet) (Fig. 2) for the presence of antibodies. We detected antibodies against the nucleoprotein (NP) in 2 snowy sheathbill samples (Fig. 3).

### Diversity and composition of the snowy sheathbill virome.

**FIG 4:**
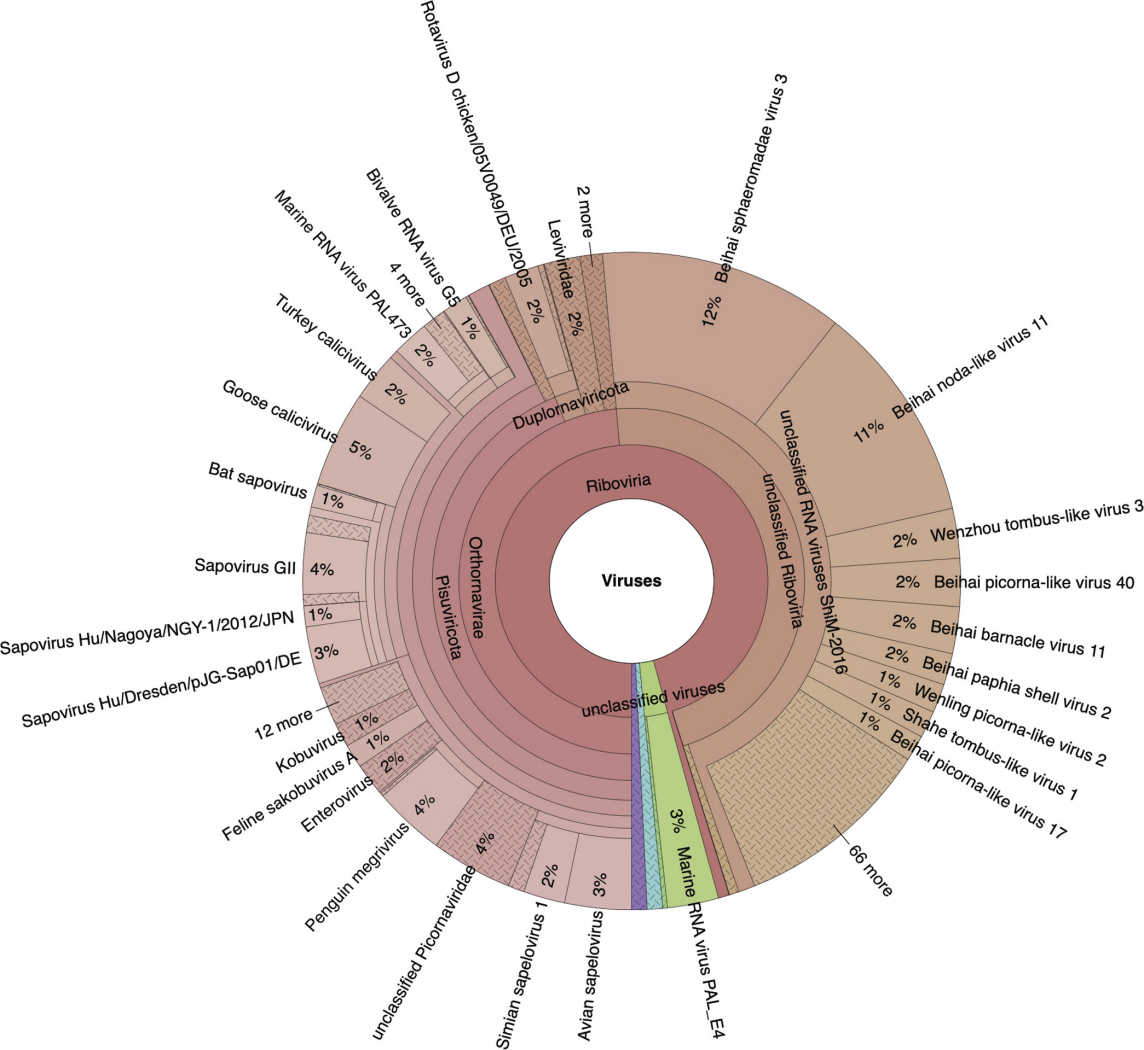
Krona plot representing an overview of all viral sequences identified by massively parallel sequencing. Taxa with >0.009% representation among the analyzed reads are shown in this Krona chart. Their names are included along with their percentages of representation. The relative abundance of viral sequences is displayed as follows: inner circles represent higher taxonomic ranks, and more detailed taxonomic ranks (up to the species level) are presented in the outer circles. An interactive version of this chart is available on the Krona website (see the URL listed in Materials and Methods). Navigational controls are at the top left, and details of the selected node are at the top right. The chart is zoomed in to place the “Viruses” domain at the root.

**FIG 5:**
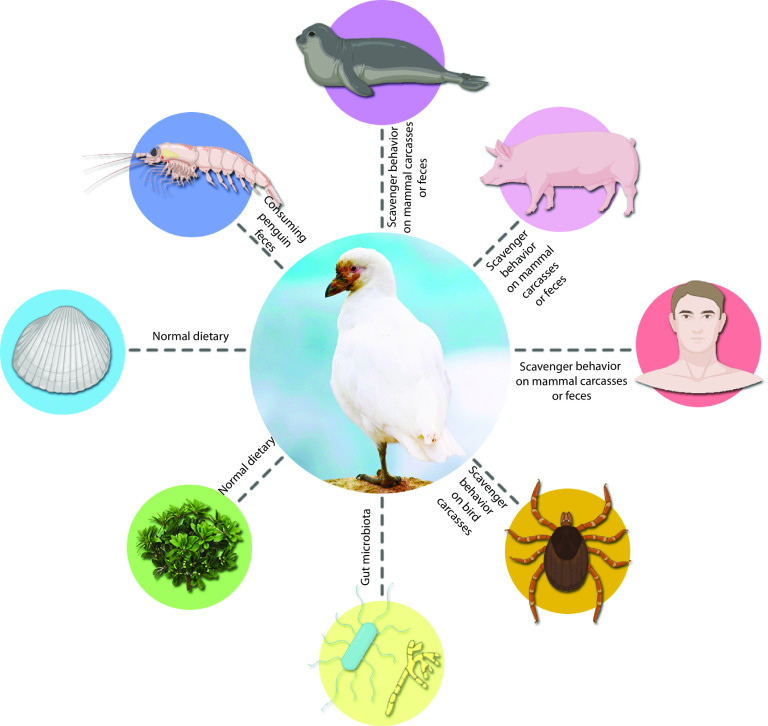
The snowy sheathbill as a sentinel of Antarctic viral diversity. Here, we display how the snowy sheathbill’s opportunistic feeding behavior can influence its harbored viral diversity and its likely source.

In order to obtain a fuller and more detailed picture of the viral diversity harbored by this endemic bird species, we characterized the snowy sheathbill viromes collected from two Antarctic locations (Fig. 4; see Fig. S1 in the supplemental material). Sequencing yielded 18,259,501 usable reads, of which 1,817,608 were of a viral origin. This allowed us to assemble 971 viral contigs of between 8,500 and 200 nucleotides (nt), with 142 hits associated with known viruses. The snowy sheathbill’s scavenger behavior is best portrayed by considerable viral diversity (Fig. 5 and Fig. S1); the library contained reads matching avian, human, nonhuman mammalian, shrimp, insect, plant, and bacterial viruses (Fig. 5). Most matches were plausible considering the snowy sheathbill’s lifestyle, and they are displayed at a viral family resolution (Fig. S1) and then up to the species level (Fig. 4), with further identity information being provided by BLAST analysis (see Table S2 in the supplemental material).

Totals of 17 viral families and 4 groups of unclassified viruses were identified (Fig. 4 and Fig. S1). The snowy sheathbill library was enriched primarily in *Ackermannviridae*, *Caliciviridae*, *Parvoviridae*, *Myoviridae*, *Nimaviridae*, and *Picornaviridae*. Fig. 4 also details the classifications of the different viral categories with further species resolution and confirms most of our findings.

Although the identification of multiple viruses was possible by sequencing (Fig. 4 and Fig. S1), we focused on viruses with potential zoonotic risk and human or mammalian viruses. Furthermore, the identities of the potential hosts for these viruses are further clarified in Table S2.

**FIG 6:**
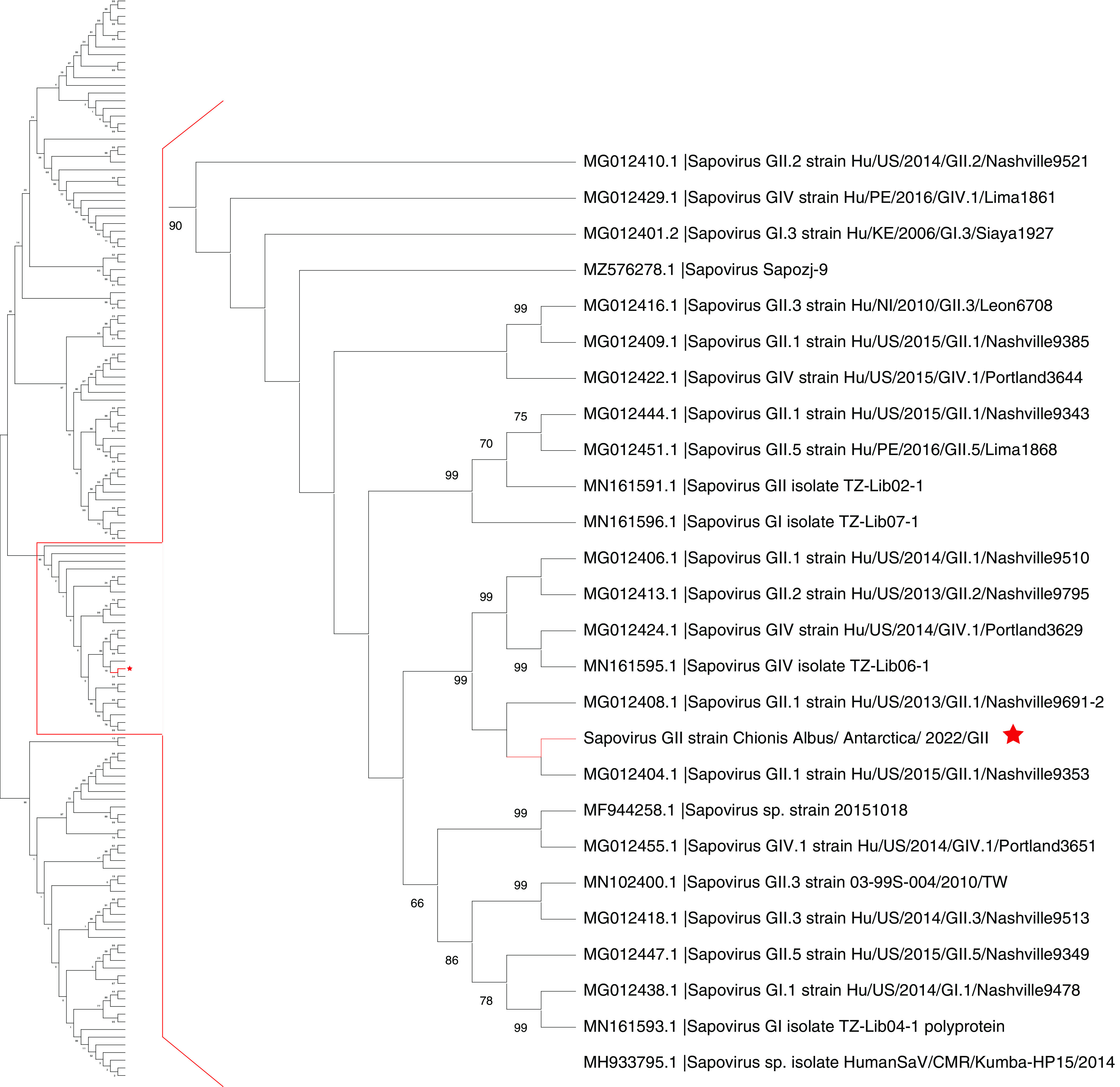
Phylogenetic tree including members of the *Sapovirus* genus. Sequence comparison was performed using 141 sequences from the *Sapovirus* genus; the sequence found in snowy sheathbills is highlighted by a red star. Phylogenetic distances were calculated using the Kimura 2-parameter method. A bootstrap analysis with 1,000 replicates was applied, and numbers indicate bootstrap values. All GenBank accession numbers are included in Tables S1 and S2 in the supplemental material. (A) Complete phylogenetic tree; (B) zoomed-in view of the clade including the newly identified sapovirus sequence.

We detected a partial human sapovirus genome with 75.0% identity to a sapovirus of North American origin (Fig. 6 and Table S1). Both methods of taxonomic classification identified this sequence. A likely human gammaherpesvirus with 98.9% identity to a Chinese herpesvirus variant strongly associated with nasopharyngeal carcinoma was also detected (Table S1). Similarly, this virus was also identified by both methods, but the visual resolution of the chart does not display the above-mentioned herpesvirus. Finally, a partial sequence from a picorna-like virus with 100% identity to a unique Antarctic fur seal virus described in 2017 was found in the data set (Table S1). Phylogenic analyses of the identified gammaherpesvirus and picorna-like virus were not pertinent since the assembled contigs were too well conserved between different species to resolve any branches. Instead, we used their respective highest-similarity hits found in the NCBI nt database, and we considered the E value as the first quality filter for the BLAST search results. Hits with an E value of <0.0001 are considered good hits for homology matches (Table S1).

## DISCUSSION

An increase in fieldwork efforts/expeditions paired with improvements in methods dedicated to the screening of microbial diversity through massively parallel sequencing has accelerated the discovery of novel pathogens in Antarctica, including bacteria and viruses (4, 5, 7, 10). However, almost all research dedicated to the characterization of the microbial diversity in Antarctic bird species has focused on penguin species (3, 10, 11). This is probably due to their high level of representation among Antarctic birds (2). Some studies have sampled scavenger species such as the snowy sheathbill, but the number of individuals was usually low, and viruses were not always targeted, certainly not on a metagenomic scale (10, 12). This encouraged us to assess whether a bird species such as the snowy sheathbill could provide us with a complementary picture of viral diversity using targeted surveillance and metatranscriptomic methods (11, 13). Furthermore, the opportunistic feeding behavior associated with this particular bird species provided an opportunity to gain improved insights into Antarctic viral diversity (2, 6). To our knowledge, this is the most abundant sampling of snowy sheathbills for a viral screening study so far (Fig. 2). In addition to targeted viral screening, the snowy sheathbill virome was used to assemble 971 viral contigs of between 8,500 and 200 nt, with 142 hits associated with known viruses. Interestingly, a high percentage of contigs showed no significant identity, suggesting potentially new highly divergent viruses in these assembled contigs. Considerable viral diversity was found, with representatives from 17 viral families and 4 groups of unclassified viruses being identified in the studied snowy sheathbill pool (Fig. 6 and Fig. S1). Moreover, we identified sequences likely related to viruses from pigs, seals, and humans in the virome of this scavenger bird (Fig. 5), although it should not be neglected that undersampling may have affected the diversity reported here. These data support the idea that scavenger birds from Antarctica provide an opportunity to study the diversity of viruses in this region. Importantly, this is, to our knowledge, the most diverse virome described from an Antarctic bird species so far (14).

Recently, a study describing the *Pygoscelis* genus virome suggested that flying bird species act as potential vectors for the transfer of pathogens among different penguin colonies separated by distances of around 200 km (11). A common example of viruses associated with penguin colonies is avian paramyxoviruses; these viruses were first described in birds from the Antarctic in 1970 using a serological approach (15). Later on, additional studies revealed the presence of avulaviruses in penguins using PCR and transcriptomic techniques (5, 15, 16). We detected paramyxoviruses in 18 samples taken from two locations in different years. This strongly suggests that flying bird species, particularly scavengers, are potential vectors and reservoirs for viruses, likely without experiencing disease. This could also entail that paramyxoviruses are widespread in these populations, which would become evident through larger screens. It should be noted that all sampled individuals were evaluated by a veterinarian and showed no clinical signs of disease.

Similar to what has been observed previously for other scavenger bird species, there are few reports describing snowy sheathbill individuals with viral infections. This is likely due to a potent immune system and/or a lower stomachic pH killing the viruses (17–19). Here, we present the first isolation of a paramyxovirus in snowy sheathbills (Table 2). We detected avulaviruses 18 and 19; however, neither sample was positive for avulavirus 17, and one sample was not positive for avulavirus 17, 18, or 19 or NDV, suggesting a potentially novel Antarctic paramyxovirus. Extensive characterization of these paramyxoviruses after isolation is under way in our laboratory. Note that snowy sheathbills shared a habitat with Antarctic penguins at the time of avulavirus 17, 18, and 19 detection, suggesting the probable circulation of the virus.

**TABLE 2:** Primers used in this study together with their targets, target lengths, cycle numbers, and corresponding references^*a*^

Primer set	Direction	Sequence	Length (bp)	Target	Annealing temp (°C)	No. of cycles	Reference
IZS	FW	5′-CDCAYGARTTYTGYTCNCARC-3′	180	RdRp	45	50	45
	RV	5′-RHGGRTANGCRTCWATDGC-3′					

Gentoo	FW	5′-CCCACTAACAAAGGTAGATC-3′	270	RdRp	54	45	This study
	RV	5′-CAGAGCAATGGTCGTCTT-3′					

PMX	FW	5′-GARGGIYIITGYCARAARNTNTGGAC-3′	121	RdRp	41	35	46
	RV	5′-TIAYIGCWATIRIYTGRTTRTCNCC-3′					

APMV1	FW	5′-AGTGATGTGCTCGGACCTTC-3′	121	M	41	40	47
	RV	5′-CCTGAGGAGAGGCATTTGCTA-3′					

AV-17	FW	5′-TTRTCATCCTACTCTACCTAT-3′	281	L	45	45	This study
	RV	5′-AGGTCATTCATATAGAGTGT-3′					

AV-18	FW	5′-GCATCAAGTAAGCAATATCT-3′	310	L	45	45	This study
	RV	5′-CTCAAGAAGAGTCATTATGTAA-3′					

AV-19	FW	5′-GGTCACAGATTCTACAAC-3′	395	L	45	45	This study
	RV	5′-GCTCAGTCCTACTTATTATTG-3′					

Inf-A	FW	5′-GACCRATCCTGTCACCTCTGAC-3′	105	M	60	40	48
	RV	5′-AGGGCATTYTGGACAAAKCGTCTA-3′					
	Probe	5′-FAM-TGCAGTCCTCGCTCACTGGGCACG-BHQ1-3′					

a FW, forward; RV, reverse; RdRp, RNA-dependent RNA polymerase; FAM, 6-carboxyfluorescein; BHQ1, black hole quencher 1.

In contrast to other Antarctic bird screenings by PCR on fecal samples from snowy sheathbills (3) and massively parallel sequencing studies on penguins that have shown the presence of influenza A virus (IAV), we did not detect the above-mentioned virus using either of these techniques. We believe that this could be due to our sample size or the time when the samples were taken (December 2020 and January 2022). Nevertheless, we detected 2 individuals with antibodies against IAV, suggesting IAV infection at a different period of its cycle (Fig. 3). Antibodies to IAV have been detected previously in a southern giant petrel that spent its nonbreeding season over Argentina and the Falkland/Malvinas Islands, supporting the general idea that migratory animals might be contaminated by IAVs during their nonbreeding season (20). Note that the test used for IAV detection has been validated for domestic chickens, turkeys, ducks, and geese according to the manufacturer’s instructions. In addition to this, Claes et al. (21) previously tested the same kit on mallards, mute swans, Canadian geese, and Peking ducks. These data suggest that the kit is relatively plastic in terms of its application to other species. It should also be noted that the NP targeted in this assay can vary between species. Considering this, we think that the most likely outcome is several false-negative results, although this is merely a hypothesis. It should be noted that this is currently the gold-standard test for Antarctic penguins.

Of relevance was the detection and isolation of avian coronaviruses. Until now, in Antarctica, avian coronaviruses were detected only through antibodies or by next-generation sequencing (NGS) and only in penguins (3, 11, 22). We were able to isolate the virus from snowy sheathbills for molecular characterization and the identification of its potential zoonotic impact. This work is ongoing in our laboratory.

The habitats and foraging and nesting habits of bird species are likely to impact the risk of the acquisition of pathogens infectious to them and other species (23). Birds foraging close to livestock show a higher incidence of the presence of enteric pathogens than birds foraging away from such sites (24, 25). Bird species foraging in anthropic sites such as rubbish dumps have a higher likelihood of exposure to zoonotic pathogens (23). Therefore, the characteristics of the environment and the habits of a given bird species affect colonization by different microorganisms (e.g., bacteria and viruses). Obligate scavenger birds such as black vultures, a bird common in rubbish dumps, can be used to detect pathogens such as Salmonella enterica and Chlamydia psittaci as they could act as potential dispersers and reservoirs of these pathogens (14). Their role as a sentinel of pathogen diversity in rubbish dumps was therefore proposed (14). Studies on other obligate scavenger species such as the Andean condor have also supported the idea that understanding the factors involved in the circulation and emergence of pathogens in these birds is key to preserving ecosystems and human health, highlighting their value for surveillance (26). Generally, it is considered that the scavenger lifestyle amplifies the exposure of such bird species to a variety of pathogens due to potential contact with infected prey or carcasses, hence the interest in them as potential sentinels. This has also been observed for additional bird species that exhibit scavenger behaviors, such as corvids, owls, and falcons, and different studies have confirmed the detection of diverse viral pathogens in them (6, 27–29). The focus on snowy sheathbills in this study offers a unique opportunity for the intersection of their scavenger behavior and their unique habitat, providing unprecedented insight for viral surveillance.

One of the most interesting findings was the identification of several viruses related to known mammalian sequences in the samples (Fig. 4 and 6; see also Table S1 in the supplemental material). We highlight one picornavirus similar to one described previously in Antarctic fur seals (Arctocephalus gazella) (Table S1) (30). The presence of such mammalian viruses (16, 31, 32) in snowy sheathbills is likely a result of their scavenger behavior, therefore also supporting the idea that scavengers are appropriate monitors/sentinels of Antarctic virus circulation/introduction. Moreover, we detected a virus similar to human sapovirus GII. Such viruses belong to the *Caliciviridae* family and are important causative agents of acute epidemic gastroenteritis in humans (33) (Fig. 5 and 6 and Fig. S1). Human infection occurs via contaminated food or water, and the respective diseases are therefore designated foodborne diseases (34). Recently, the presence of human sapoviruses and other gastrointestinal viruses such as noroviruses in farm animals has attracted increasing attention. This is due to the fact that transmission from animals to humans and vice versa would have far-reaching consequences for epidemiology and food safety (33, 34). Ultimately, this finding suggests the circulation of human viruses on the Antarctic continent. The impact that this virus could have on Antarctic fauna is unknown and complex to estimate. The identification of several human-associated viruses in our study could be considered an indication of contamination. However, we consider that the techniques and precautions applied for sample collection and preparation make contamination rather unlikely. The same sample treatment for viral enrichment was applied in a bat virome study, with no biases concerning human-associated viruses observed (35). It should also be mentioned that at least one of the sampled populations in this study is in contact with humans (General Bernardo O’Higgins military base). Humans have shared this particular environment with snowy sheathbills for 75 years, which likely has an impact. In addition to this, one of the human-associated viruses identified in this study was a gammaherpesvirus with 98.9% identity to a Chinese herpesvirus. The level of identity to this geographically distant isolate makes contamination an unlikely option. We consider that these arguments collectively make contamination an unlikely event.

The gammaherpesvirus found in our samples was closely related to a specific Chinese herpesvirus that may cause nasopharynx cancer in humans (36) (Table S1). Epstein-Barr virus infection and environmental exposure to specific substances are considered risk factors, even though this condition is endemic in some Asian areas where a genetic predisposition for oncogenesis has been established (36). The identification of such a virus is surprising and concerning, particularly because the consequences or the probability of transmission to marine mammals is unknown. Its origin is a puzzling question that is yet to be solved.

We highlight the need for viral surveillance in remote regions, a goal that possibly can be achieved by monitoring scavenging seabirds. We found considerable viral diversity in snowy sheathbills (Fig. 5), likely associated with their opportunistic diet. We show evidence of several potential human virus introductions in Antarctica and showcase the snowy sheathbill as a candidate sentinel of viral diversity. We highlight the importance of continuous avian surveillance, including other Antarctic scavenger birds; this will be critical for elucidating and better understanding the mechanisms of virus introduction and circulation in Antarctica.

## MATERIALS AND METHODS

### Sample collection.

Samples were collected on two separate trips during Chilean Antarctic Expedition 57 (ECA57) and Chilean Antarctic Expedition 58 (ECA58). ECA57 took place from 15 to 18 December 2020 on Nelson Island, more specifically on the Harmony Point coast (62°18′0″S, 59°13′0″W). ECA58 took place from 13 January to 9 February 2022 in the Antarctic Peninsula Isabel Riquelme Islet and Kopaitic Island (on the Duroch Islands) (63°19′5″S, 57°53′55″W) (Fig. 2). Harmony Point is an island considered to be devoid of the presence of humans. Sampling during ECA57 was performed around the island’s penguin colony’s nesting grounds. Sampling during ECA58 was performed at the General Bernardo O’Higgins military base’s penguin colony site. This is in contrast to ECA57 due to the direct interactions of the sampled subjects with humans. During 2020, a total of 124 direct environmental samples of Antarctic avifauna were collected, of which 22 samples were identified for the following specific species: giant petrel (*Macronectes giganteus*) (*n* = 8), snowy sheathbill (*Chionis albus*) (*n* = 7), Weddell seal (*Leptonychotes weddellii*) (*n* = 6), and Antarctic shag (*Leucocarbo bransfieldensis*) (*n* = 1). These samples were collected with sterile swabs and placed into 1.5-mL Eppendorf tubes with viral transport medium (VTM) from VQIR (catalog number 611901) (7). The samples were kept at ambient temperature in a cooler (near 0°C) before being stored at −80°C. It should be noted that during this expedition, we were evacuated from Antarctica due to a coronavirus disease (COVID) outbreak, which impacted our sampling capacity/numbers. Serum samples were taken from the medial metatarsal vein. During ECA58, a total of 105 samples were collected: cloacal swabs and serum samples (*n* = 14) and environmental samples (which correspond to fecal dropping samples) (*n* = 33) from snowy sheathbills (*Chionis albus*) and environmental samples from Antarctic terns (*Sterna vittata*) (*n* = 33) and chinstrap penguins (*Pygoscelis antarctica*) (*n* = 25) were taken between three islands (Nelson, Isabel Riquelme Islet, and Kopaitic Island). Serum samples were taken from the medial metatarsal vein. Samples were stored at −80°C. All sampled individuals were examined by a veterinarian.

### Screening for anti-influenza A virus antibodies.

An influenza A virus antibody test kit blocking enzyme-linked immunosorbent assay (ELISA) was used according to the manufacturer’s instructions (AI MultiS-Screen, catalog number 99-53101; Idexx).

### RNA extraction.

The samples were homogenized in VTM. After decanting the particles present in the sample, 60 μL of the supernatant was taken, and pools were prepared for each of 5 samples with 100 μL of VTM. The pools were clarified by subsequent centrifugation at 8,000 × *g* for 10 min at 4°C and stored at −80°C. RNA was extracted from 150 μL of the supernatant obtained in the previous step, using TRIzol reagent (Invitrogen) based on the Chomczynski-Sacchi method (37), according to the manufacturer’s instructions. The RNA obtained was resuspended in 30 μL of nuclease-free water and stored at −20°C. The RNA concentration was analyzed by absorption using a Synergy HTX multimodal reader (Agilent Technologies, USA).

### Targeted screening.

Screening for coronavirus, including the Antartic1 replicase (this study); paramyxovirus (PMX); and influenza virus was performed using Brilliant III Ultra-Fast RT-PCR master mix (Agilent Technologies, USA).

Concerning the sensitivity of our screen for influenza virus detection, degenerate primers were used for RT-quantitative PCR (RT-qPCR) screening. We followed WHO recommendations, considering a positive result until cycle 38. Coronavirus and paramyxovirus detection was performed by RT-PCR, and positive identification was determined by the presence or absence of a band of the expected size in an agarose gel.

### Virus isolation.

For the amplification and isolation of coronaviruses and paramyxoviruses, passages were made in 8- to 10-day-old specific-pathogen-free embryonated eggs obtained from the Institute of Public Health (ISP), Chile. The inoculated sample was prepared from 100 μL of the supernatant obtained after the homogenization and clarification of the samples (pools and/or individual samples) and 100 μL of VTM, inoculating 100 μL per egg in duplicate. Finally, the inoculated eggs were incubated in a humid environment at 37°C, where they were left for 48 h. Once the incubation time was over, the eggs were left at 4°C for a minimum of 4 h for euthanization. After incubation, the allantoic fluid was collected, obtaining between 2 and 3 mL per egg. The allantoic fluids obtained were stored at −80°C.

### Viral purification and concentration.

Ultracentrifugation was performed based on a protocol described previously by Fumian et al. (38). Six snowy sheathbill fecal samples were taken, homogenized in 1 mL of VTM, and clarified at 7,000 × *g* for 10 min at 4°C. The supernatant obtained was recovered and centrifuged at 13,000 × *g* for 30 min at 4°C. This new supernatant was transferred to a cryogenic tube, and the volume was made up to 1 mL. Samples were centrifuged at 30,000 × *g* for 3 h 15 min. Next, 900 μL of the supernatant was removed, resuspended in 150 μL of 0.25 N glycine buffer (pH 9.5), and incubated on ice for 30 min. The suspension was neutralized by the addition of 150 μL of 2× phosphate-buffered saline (PBS) (pH 7.2). The supernatant, rich in viral particles, was centrifuged at 12,000 × *g* for 15 min at 4°C, and viruses were recovered by centrifugation at 30,000 × *g* for 3 h 15 min at 4°C. Finally, the supernatant was discarded, and the pellet was resuspended in 200 μL of 1× PBS (pH 7.2). DNases and RNases were added to a final concentration of 10 U. The samples were stored at −80°C until use.

### Depletion, library preparation, and NGS.

After total RNA enrichment for viral particles by centrifugation, total RNA was depleted for rRNA using the Illumina Ribozero Plus kit with a custom protocol in Magelia (Inorevia, Paris, France). After bioinformatic verification of probe compatibility for bacterial/bird selection, 2 μL of total RNA was used in the instrument for the fully automated molecular enrichment of viral sequences. After depletion, total RNA was run in the Bioanalyzer 2100 system using a nano-RNA chip to determine rRNA contamination. Next, the Illumina total stranded RNA protocol for library preparation was performed using approximately 10 ng of depleted RNA. Library preparation yielded 20 nM high-quality sequenceable material, which was then run in a NextSeq 500 Mid Output kit v2 (150 cycles) cartridge. This yielded a total of 36,519,002 sequences in pairs.

### Bioinformatic analyses.

Read quality control was evaluated using FastQC v0.11.9, and read preprocessing was performed with fastx-toolkit v0.0.13 using the fastx_trimmer, fastx_artifacts_filter, and fastx-clipper tools. Orphaned reads were separated from the total sample using the makepairs package of pairfq v0.17.0. The Kraken2-bracken protocol was implemented for taxonomic read assignment. The sample assembly was generated using SPAdes genome assembler v3.13.0 (metaSPAdes mode). To avoid overestimation, only contigs with a minimum size established at 200 nucleotides were selected. For contig clustering and nonredundancy, CD-HIT software was used, covering the nonrepresentative sequences at 80%.

Bowtie2 v2.4.2 was used to recapture clean reads mapped to the contig sequence database. Sample processing was implemented in Samtools v1.13. Read quantification was performed using samtools view -c -F 260.

A BLAST analysis was also performed on the contigs using the NCBI BLASTN algorithm. A supplemental table was generated with the top hits for the generated contigs with an E value cutoff of E−08. This enabled us to clarify the potential host of origin for the identified viruses.

As a complement, the reads were run using the Kaiju Web tool as previously described (35). This enabled us to produce a summary file with the number of reads assigned per taxon, which was then loaded into Krona (39) for interactive visualization. The interactive plot can be found at https://kaiju.binf.ku.dk/output/10391-7724754407/krona.html?dataset=0&node=3062&collapse=true&color=false&depth=13&font=11&key=true.

### Oxford Nanopore MinION sequencing.

The coronavirus isolate was sequenced using the Oxford Nanopore MinION platform. Briefly, starting with 250 ng of high-molecular-weight (50-kbp) double-stranded DNA (dsDNA) as the input, fragments with an average distribution of 11 kbp were generated by the use of a g-Tube for DNA shearing (Covaris) and centrifugation at 2,000 × *g* for 1 min. Using the NEBNext FFPE (formalin-fixed, paraffin-embedded) DNA repair mix (New England BioLabs) and NEBNext Ultra II end repair/dA-tailing module (New England BioLabs) reagents and according to the instructions provided by Oxford Nanopore Technologies, the ends of the DNA were repaired, to which a poly(A) tail was attached. The repaired DNA was purified using AMPure XP (Beckman Coulter) according to the manufacturer’s instructions and using a 2× volume with respect to the sample. For all subsequent washes, this volume ratio of beads to sample was maintained in order to maximize the DNA concentrations recovered at the expense of fragment size. According to the protocol provided by Oxford Nanopore Technologies, the NEBNext quick ligation module reagent (New England BioLabs) was used for the ligation of the adapter to the nucleotide sequence. For the following steps, the library preparation kit for MinION SQK-DCS 109 (Oxford Nanopore Technologies) was used, according to the company’s instructions. The library was loaded onto a MinION flow cell (R9.4.1). For sequencing, the minKNOW v3.4.5 program was used (40).

### Genome assembly.

The quality of the reads was assessed using NanoPlot v1.23.1 (41). Adapter sequences were removed with Porechop v0.2.4. (https://github.com/rrwick/Porechop) using a 10,000-read alignment. Reads were quality cut using the Nanofilt v2.3.0 tool (41). Assembly was performed using Unicycler v0.4.8-beta (40) in long-sequence mode. Finally, the quality of the assembly was evaluated using the QUAST v5.0.2 tool (42).

### Sapovirus phylogenetic analyses.

We compiled a sapovirus database with 141 sequences (Fig. 5; see also Table S1 in the supplemental material). Alignments were performed using MUSCLE (http://www.drive5.com/muscle/) (43). Phylogenetic trees were constructed with MEGA6 (http://www.megasoftware.net) and IQ-TREE on the IQ-TREE Web server (http://www.cibiv.at/software/iqtree/) (44) by using the maximum likelihood (ML) method. The robustness of the tree topologies was assessed with 1,000 bootstrap replicates. Phylogenetic trees were constructed using ML inference with the Kimura 2-parameter nucleotide substitution model.

### Deltacoronavirus phylogenetic analyses.

We compiled a coronavirus database with 78 sequences (Table S2). Alignments were performed using MUSCLE (http://www.drive5.com/muscle/) (43). Phylogenetic trees were constructed with MEGA6 (http://www.megasoftware.net) and IQ-TREE on the IQ-TREE Web server (http://www.cibiv.at/software/iqtree/) (44) by using the ML method. The robustness of the tree topologies was assessed with 1,000 bootstrap replicates. Phylogenetic trees were constructed using ML inference with the general time-reversible (GTR) parameter nucleotide substitution model.

### Ethics statement.

The study described here was approved by the Ethics Committee of the Faculty of Medicine at the Universidad de Chile (approval number 20349-med.uch).

### Data availability.

The data supporting the findings of this study can be found in this article and its supplemental material. All Illumina sequencing data used for this RNA sequencing (RNA-seq) study have been submitted to the Sequence Read Archive (SRA) under BioProject accession number PRJNA904746.
