# Supplementary material for: Scavengers as Prospective Sentinels of Viral Diversity: the Snowy Sheathbill Virome as a Potential Tool for Monitoring Virus Circulation, Lessons from Two Antarctic Expeditions
Source: Microbiol Spectr. 2023 May 25;11(3):e03302-22. doi: 10.1128/spectrum.03302-22 (PMC10269608; doi:10.1128/spectrum.03302-22)
Supplement: Supplemental file 1 — Table S1. Download spectrum.03302-22-s0001.pdf, PDF file, 0.5 MB [file spectrum.03302-22-s0001.pdf]

Virus	Qseqid	sseqid	pident	length	mismatch	gapopen	qstart	qend	sstart	ssend	evalue	bitscore	qcovus
Sapovirus	Contig_6	MG515475.1	75.936	187	43	2	2693	2878	2818	3003	7.67e-16	95.3	3
	Contig_6	MG515474.1	75.936	187	43	2	2693	2878	2818	3003	7.67e-16	95.3	3
	Contig_6	AB630068.3	75.789	190	38	3	2693	2878	2818	3003	3.57e-14	89.8	3
	Contig_6	MG515473.1	75.401	187	44	2	2693	2878	2818	3003	3.57e-14	89.8	3
	Contig_6	MF944258.1	72.792	283	65	7	2693	2969	2819	3095	4.61e-13	86.1	4
Herpesvirus	Contig_155	MG934417.1	90	60	4	2	433	491	246	188	2.06e-11	76.8	10
	Contig_169	MH590442.1	82,151	437	78	0	26	462	168524	168088	1.00e-118	433	82
	Contig_313	MH590571.1	98,928	373	4	0	2	374	36060	36432	0.0	671	99
	Contig_313	MH590409.1	98660	373	5	0	2	374	157846	158218	0.0	667	99
	Contig_536	MH590442.1	92,058	277	22	0	2	278	168176	168452	1.79e-118	431	99
	Contig_573	MH590571.1	98,881	268	3	0	2	269	34989	34722	6.04e-133	479	99
	Contig_573	MH590409.1	98,507	268	4	0	2	269	156775	156508	7.81e-132	475	99
	Contig_601	MH590442.1	85824	261	37	0	1	261	168353	168093	2.27e-82	311	99
	Contig_772	MH590409.1	96,053	228	9	0	2	229	158182	158409	5.28e-103	379	99
	Contig_772	MH590571.1	96,721	61	2	0	2	62	36396	36456	1.28e-19	102	27
	Contig_845	MH590376.1	98,758	161	2	0	57	217	36670	36830	8.70e-76	289	73
	Contig_845	MH590571.1	96,721	61	2	0	3	63	34034	33974	3.38e-20	104	28
	Contig_927	MH590571.1	96,635	208	7	0	1	208	34432	34225	1.34e-93	348	99
	Contig_927	MH590409.1	98,276	58	1	0	1	58	156218	156161	1.15e-19	102	28
	Contig_943	MH590571.1	99,515	206	1	0	2	207	35294	35499	6.08e-102	375	99
	Contig_943	MH590409.1	99,029	206	2	0	2	207	157080	157285	2.83e-100	370	99
	Contig_948	MH590571.1	99,024	205	2	0	2	206	35313	35109	1.01e-99	368	99
	Contig_948	MH590409.1	98,537	205	3	0	2	206	157099	156895	1.31e-98	364	99
Picorna-like virus	Contig_551	NC_035110.1	100.000	274	0	0	2	275	1849	2122	2.85e-141	507	99
	Contig_551	KY926885.1	100.000	274	0	0	2	275	1849	2122	2.85e-141	507	99
	Contig_551	NC_029307.1	99.273	275	2	0	2	276	1891	2165	1.72e-138	497	99
	Contig_551	KT727024.1	99.273	275	2	0	2	276	1891	2165	1.72e-138	497	99

Table S1: Contig assemble with their respective similar hits from Viruses nt NCBI database: yellow indicate sapovirus contig assembly, green indicates herpesvirus contig assembly and blue indicate fur seal picornavirus contig assembly. E-value is used as a first quality filter for the BLAST search result. Hits with an E-value smaller than 0.0001, includes database matches of very high quality considered as good hits for homology matches.
